# A Twisted Case Of Jaundice

**DOI:** 10.7759/cureus.6683

**Published:** 2020-01-16

**Authors:** Hung Hoang, Tara Norris

**Affiliations:** 1 Internal Medicine, Methodist Health System, Dallas, USA

**Keywords:** jaundice, obstructive jaundice, diaphragmatic hernias, biliary obstruction

## Abstract

Diaphragmatic hernias (DH) may be congenital or acquired in origin. Those causing obstructive jaundice in the elderly are extremely rare but can occur. These patients may present with painless jaundice, early satiety, and weight loss due to biliary tract obstruction and stomach compression by the hernia. Therefore, clinicians should consider an anatomic anomaly when evaluating patients with jaundice. Here, we report the case of a 71-year-old female, with a medical history of hypertension and chronic obstructive pulmonary disease, who presented with jaundice. The patient was found to have dilation of the common bile duct due to external mechanical compression of abdominal organs from a DH. Because the patient had poor functional status and multiple comorbidities, the risks of surgically correcting the hernia outweighed the benefits. The patient instead received a biliary decompression and stent, and her jaundice significantly improved.

## Introduction

With biliary obstruction, patients often present with clinical signs of yellowing skin, known as jaundice. Typically, laboratory data demonstrates conjugated hyperbilirubinemia and imaging studies show dilation of the common bile duct. Diaphragmatic hernia (DH) is a herniation of abdominal viscera into the thorax through a diaphragmatic defect. A typical clinical presentation of DH can vary from asymptomatic to respiratory failure [1] and bowel obstruction [2]. Extra-hepatic obstruction due to DH in adults is rare [3-4]. We present such a case of extra-hepatic obstructive jaundice due to DH.

## Case presentation

A 71-year-old woman with a past medical history of hypertension and chronic obstructive pulmonary disease presented to the hospital after her granddaughter noticed two days of worsening jaundice. The patient reported a one-week history of fatigue, early satiety, anorexia, nausea, and vomiting. She also noted tea-colored urine recently, and an unintentional 30-pound weight loss during the prior eight months. She had no history of trauma, abdominal pain, fever, or chills. Her surgical history included tubal ligation many years prior.

At the time of admission, the patient had diffuse jaundice. Her vital signs were normal as was the remainder of the physical examination. Total bilirubin was 13.4 mg/dL, with a direct bilirubin of 11.8 mg/dL. Her liver function tests were also notable for aspartate aminotransferase (AST) of 176 U/L, alanine aminotransferase (ALT) of 89 U/L, and alkaline phosphatase of 1128 U/L. 

**Figure 1:**
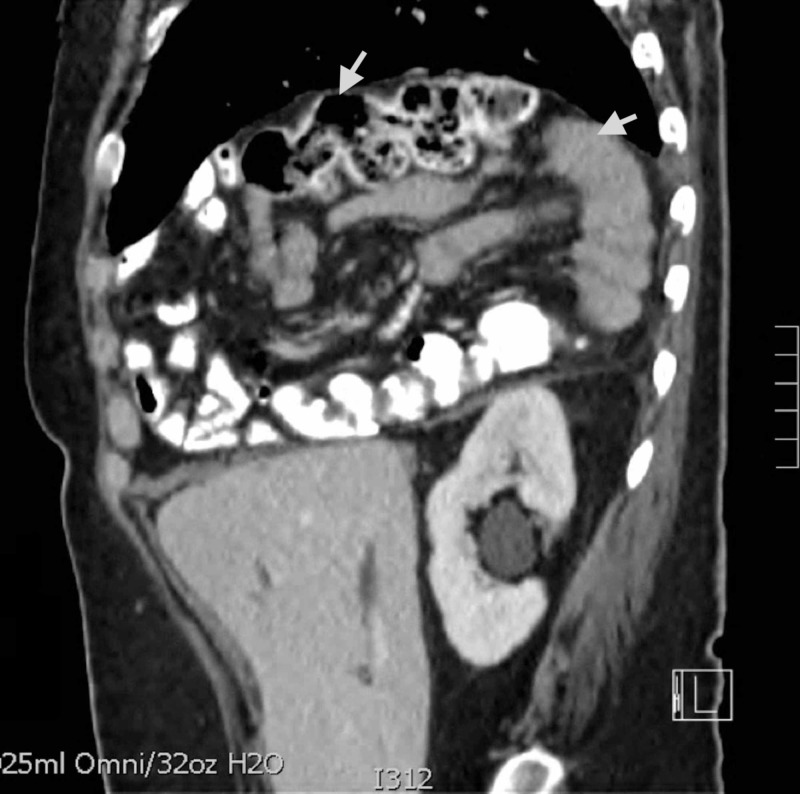
Large diaphragmatic hernia with the stomach, pancreas, entire small bowel and most of the colon located in the thorax

**Figure 2:**
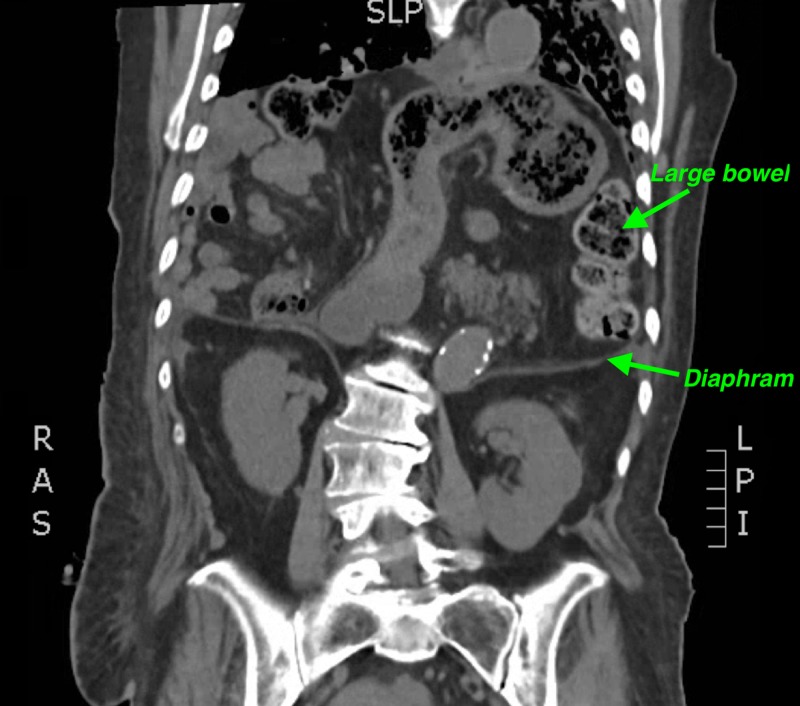
Bowel in the left thorax

Abdominal ultrasound demonstrated a dilated common bile duct at 8 mm, as well as intrahepatic ductal dilation. Magnetic resonance cholangiopancreatography (MRCP) demonstrated stomach, pancreas, small bowel and most of the colon above the diaphragm, and it revealed abnormal orientation of the extrahepatic bile ducts extending superiorly to the duodenum located in the thorax. Computed tomography (CT) of thorax, abdomen, and pelvis revealed a large DH, and it confirmed the presence of pancreas, small bowel, and colon in the thorax (Figures 1-2).

Surgical repair of the defect was considered, but the patient’s poor functional status and increased surgical risk were determined to outweigh the benefits. Instead, biliary decompression via stent placement was performed percutaneously, as her anatomy was not amenable to an endoscopic approach. 

She tolerated the procedure well, and her jaundice and transaminitis subsequently resolved. Follow-up plans were made for stent replacement, as needed, for recurrence of jaundice. 

## Discussion

DH may be congenital, acquired, or even caused by trauma. The vast majority of congenital diaphragmatic hernias (CHD) present in infancy or childhood, and occur at a rate of less than 5 per 10,000 live births [5]. The incidence of CDH found incidentally on CT in adults is 0.17%, but the incidence of CHD leading to clinical presentation has not been reported [6]. While several case reports of CDH causing obstructive jaundice can be found in the pediatric literature, only two published cases could be located describing this phenomenon in adults. One case was reported in a 19-year-old female, and the other was reported in a 64-year-old female [3-4]. 

The clinical manifestations of DH can vary, from asymptomatic to respiratory or gastroenterology symptoms [1-2], or jaundice [3-4], depending on the location and severity of the diaphragmatic defect, and organ involvement. DH can be confirmed or diagnosed with ultrasonography, magnetic resonance imaging (MRI), or CT of the thorax, abdomen, and pelvis. 

Our patient reported no history of trauma, therefore, her DH was presumed to be congenital. Her weight loss, poor appetite, and early satiety were explained by the DH’s compression of the stomach and bowel in the thorax. The treatment of choice is surgical correction of the hernia to decompress biliary obstruction, and prevent bowel obstruction, ischemia and lung injury [7]. Since this patient had poor functional status with high surgical risk, temporary biliary decompression and stent placement were recommended and implemented. Her jaundice and laboratory evidence of biliary obstruction and liver injury resolved. 

(Abstract: The 2019 Annual Meeting of the Society of General Internal Medicine; 2019). https://link.springer.com/article/10.1007%2Fs11606-019-05007-5

## Conclusions

Painless jaundice with early satiety and weight loss in the elderly population most commonly raises concern for biliary obstruction due to neoplasm. These signs and symptoms can also be caused by compression of the common bile duct from a complication of a DH. Newly discovered anatomic anomalies are rare in elderly patients, but nonetheless should be considered by clinicians when evaluating patients with jaundice. Appropriate treatment can involve surgical or non-invasive techniques as highlighted in our case.
